# Readiness of Ugandan health services for the management of outpatients with chronic diseases

**DOI:** 10.1111/tmi.12560

**Published:** 2015-07-08

**Authors:** David Katende, Gerald Mutungi, Kathy Baisley, Samuel Biraro, Eric Ikoona, Robert Peck, Liam Smeeth, Richard Hayes, Paula Munderi, Heiner Grosskurth

**Affiliations:** ^1^ Medical Research Council/Uganda Virus Research Institute Uganda Research Unit on AIDSEntebbeUganda; ^2^ Ministry of HealthKampalaUganda; ^3^ MRC Tropical Epidemiology GroupLondon School of Hygiene & Tropical MedicineLondonUK; ^4^ Mwanza Intervention Trials Unit and Weill Bugando School of MedicineMwanzaTanzania; ^5^ Department of Non‐communicable Disease EpidemiologyLondon School of Hygiene & Tropical MedicineLondonUK

**Keywords:** outpatients, chronic diseases, healthcare systems, health services, sub‐Saharan Africa, Uganda, patients ambulatoires, maladies chroniques, systèmes de santé, services de santé, Afrique subsaharienne, Ouganda

## Abstract

**Objective:**

Traditionally, health systems in sub‐Saharan Africa have focused on acute conditions. Few data exist on the readiness of African health facilities (HFs) to address the growing burden of chronic diseases (CDs), specifically chronic, non‐communicable diseases (NCDs).

**Methods:**

A stratified random sample of 28 urban and rural Ugandan HFs was surveyed to document the burden of selected CDs by analysing the service statistics, service availability and service readiness using a modified WHO Service Availability and Readiness Assessment questionnaire. Knowledge, skills and practice in the management of CDs of 222 health workers were assessed through a self‐completed questionnaire.

**Results:**

Among adult outpatient visits at hospitals, 33% were for CDs including HIV *vs*. 14% and 4% at medium‐sized and small health centres, respectively. Many HFs lacked guidelines, diagnostic equipment and essential medicines for the primary management of CDs; training and reporting systems were weak. Lower‐level facilities routinely referred patients with hypertension and diabetes. HIV services accounted for most CD visits and were stronger than NCD services. Systems were weaker in lower*‐*level HFs. Non‐doctor clinicians and nurses lacked knowledge and experience in NCD care.

**Conclusion:**

Compared with higher level HFs, lower‐level ones are less prepared and little used for CD care. Health systems in Uganda, particularly lower*‐*level HFs, urgently need improvement in managing common NCDs to cope with the growing burden. This should include the provision of standard guidelines, essential diagnostic equipment and drugs, training of health workers, supportive supervision and improved referral systems. Substantially better HIV basic service readiness demonstrates that improved NCD care is feasible.

## Abstract (fr)

**Objectif:**

Traditionnellement, les systèmes de santé en Afrique subsaharienne ont mis l'accent sur les maladies aiguës. Peu de données existent sur l’état d'apprêtement des établissements de santé (ES) africains pour faire face à la charge croissante des maladies chroniques (MC), spécifiquement chroniques, les maladies non transmissibles (MNT).

**Méthodes:**

Un échantillon aléatoire stratifié de 28 ES urbains et ruraux ougandais a été interrogé pour documenter la charge de MC sélectionnées, en analysant les statistiques des services, la disponibilité des services et l'apprêtement des services à l'aide d'un questionnaire SARA modifié de l’OMS. Les connaissances, compétences et pratiques dans la prise en charge des MC de 222 agents de la santé ont été évaluées par le biais d'un questionnaire auto‐administré.

**Résultats:**

Parmi les visites ambulatoires d'adultes dans les hôpitaux, 33% étaient pour des MC dont le VIH, contre 14% et 4% dans les centres de santé de taille moyenne et petite, respectivement. Beaucoup d’ES manquaient de directives, de matériel de diagnostic et des médicaments essentiels pour la prise en charge primaire des MC; les systèmes de formation et de report étaient faibles. Les établissements de niveau inférieur référaient couramment les patients souffrant d'hypertension et de diabète. Les services VIH enregistraient la plupart des visites de MC et étaient plus préparés que les services des MNT. Les systèmes étaient moins préparés dans les ES de niveau inférieur. Les cliniciens non‐médecins et les infirmier(e)s manquaient de connaissances et d'expérience dans les soins des MNT.

**Conclusion:**

Comparés aux ES de niveau supérieur, ceux de niveau inférieur sont moins bien préparés et peu utilisés pour les soins de MC. Les systèmes de santé en Ouganda, particulièrement les ES de niveau inférieur, ont urgemment besoin d'amélioration pour la prise en charge des MNT courantes afin de pouvoir faire face à la charge croissante. Cela devrait inclure la fourniture de directives standard, l’équipement de diagnostic et les médicaments essentiels, la formation des agents de santé, la supervision formative et l'amélioration des systèmes d'aiguillage. L'apprêtement substantiellement meilleur des services VIH de base démontre que l'amélioration des soins des MNT est faisable.

## Abstract (es)

**Objetivo:**

Tradicionalmente, los sistemas sanitarios en África subsahariana se han centrado en condiciones agudas. Existen pocos datos sobre el nivel de preparación de los centros sanitarios (CS) africanos para afrontar la cada vez mayor carga de enfermedades crónicas (EC), específicamente las enfermedades crónicas no transmisibles (ECNs).

**Métodos:**

Se evaluó una muestra estratificada y aleatoria de 28 SC urbanos y rurales de Uganda, para documentar la carga de ECs mediante el análisis de estadísticas del servicio, disponibilidad de servicios y nivel de preparación de los servicios utilizando una modificación del cuestionario SARA de la OMS. Se evaluaron los conocimientos, las capacidades y las prácticas en el manejo de las ECs de 222 trabajadores sanitarios mediante un cuestionario autocompletado.

**Resultados:**

Entre los adultos que visitaron los hospitales como pacientes externos, un 33% lo hizo por ECs incluyendo VIH versus 14% y 4% en centros sanitarios de tamaño medio y pequeño, respectivamente. En muchos CSs había una falta de guías, equipos de diagnóstico y medicamentos esenciales para el manejo primario de ECs; los sistemas de formación e informes eran débiles. Los centros de menor nivel referían a sus pacientes con hipertensión y diabetes de forma rutinaria. Los servicios de VIH recibían la mayoría de las visitas por ECs y eran más fuertes que los servicios para ECNs. Los sistemas eran más débiles en CSs de menor nivel. Los sanitarios no médicos y las enfermeras no tenían ni los conocimientos ni la experiencia para la atención de ECN.

**Conclusión:**

Comparado con CS de mayor nivel, los de menor nivel están menos preparados y poco acostumbrados a la atención de ECs. Los sistemas sanitarios en Uganda, en particular los CS de menor nivel, necesitan mejorar urgentemente el manejo de ECN para afrontar una carga que va en aumento. Ello incluye contar con guías estandarizadas, equipos de diagnóstico y medicamentos esenciales, entrenamiento de los trabajadores sanitarios, supervisión de apoyo y sistemas de referencia mejorados. Unos servicios de atención básica al VIH sustancialmente mejores son la prueba de que es factible contar con una atención mejorada de las ECN.

## Introduction

Sub‐Saharan Africa (SSA) is experiencing a rapidly evolving epidemiological transition marked by an increase in chronic diseases (CDs) whilst the prevalence of classical infectious diseases is still substantial 1, 2. The burden of non‐communicable diseases (NCDs) is high and growing in both urban and rural areas 3, 4. Due to the introduction of antiretroviral treatment, HIV infection has become a manageable chronic condition 5.

Traditionally, health services in SSA have been designed to manage a high burden of acute conditions. However, Africa's primary care facilities may not be sufficiently equipped to cope with the increasing CD burden, particularly with NCDs 6, and the East African Region seems to be no exception 7, 8. Health services research related to NCDs has been identified as a priority 9, 10. To address these needs and to inform the design of an intervention programme for CD care, we conducted service surveys in health facilities in Tanzania 8 and Uganda.

The primary healthcare system of Uganda is tiered alongside the politico‐administrative organisation of the country (Table 1) and is overseen by the district health office, led by an experienced medical doctor (MD), who co‐ordinates resource distribution and staff deployment 11 to district hospitals and health centres (HCs) IV, III and II. Several districts form a region which is served by a regional hospital that can provide specialist care. HCIIs and HCIIIs which may include some private‐not‐for‐profit health facilities (PNFPs) are expected to diagnose and manage uncomplicated CD cases including diabetes mellitus (DM), hypertension, asthma and HIV infection. HCIIs which should be able to diagnose DM usually refer patients to a HCIII or higher level facility 12. The Ministry of Health (MOH) recently established an NCD department. A national NCD strategy is in preparation, and a national NCD survey has recently been completed.

**Table 1:** Description of the levels of public health service delivery and administration in Uganda

Health facility level*	Political or administrative level	Target population	Main function or infrastructural requirement(s)	Facility head/supervisor†
Regional Hospital	Region or several districts	>2 million	Specialist services, for example ophthalmology	Medical Director (e.g. MD or Public Health specialist)
District Health Office	District	500 000–2 million	Resource distribution, staffing	DHO (e.g. MD or Public Health specialist)
District or General Hospital/HCIV	District or Constituency	100 000–500 000	50–100 inpatient beds, general theatre, general laboratory	Medical Director (e.g. MD or Public Health specialist)
HCIII	Subcounty	30 000	Maternity unit, a simple laboratory	Non‐MD Clinician or Mid‐wife
HCII	Parish or several villages	5000–10 000	First‐line emergency and outpatient care	Nurse
HCI	Village	1000–5000	Outreach post, village health team	Nurse assistant or Health visitor

*HC, Health Centre; †MD, medical doctor; DHO, District Health Officer.

Adapted with permission from information sourced from Uganda MOH 11.

In this study, we describe the results of a survey of a random sample of urban and rural health facilities in central Uganda that assesses the burden of CDs, and service readiness with regard to management of these diseases.

## Methods

### Study setting

This study was performed between November 2012 and April 2013 in the districts of Wakiso and Mpigi in Central Uganda. Wakiso includes urban, peri‐urban and rural areas with a population of about 2 million 13. Mpigi lies on the shores of Lake Victoria and has a population of about 250 000 13. It comprises peri‐urban and rural areas. Main economic activities include subsistence farming, fishing and some small‐scale businesses and factories.

### Sampling

A total of 28 public or PNFP health facilities were sampled from a total of 55 facilities, all of which serve communities that participated in a population survey of CDs conducted in the project area during the same year 14. Sampling was stratified by health facility level and geographical area. The only regional hospital, situated in Entebbe Municipality, and the only two existing district town HCIVs (Wakiso and Mpigi) were purposively included. One of two existing PNFP hospitals and two of five urban HCIIIs were randomly selected. In rural areas, 10 of the 21 existing HCIIIs and 12 of 24 HCIIs were also randomly selected.

### Recruitment of health workers

All health workers who provide care for CDs were invited for interview. A list of eligible health workers was obtained from heads of facilities or departments. Health workers included MDs, non‐MD clinicians (clinical officers), nurses, midwives and nurse (clinic) assistants. Several visits were made to each facility to interview any staff absent on the first day.

### Data collection

Data were collected by a team of MDs, non‐MD clinicians and nurses under the direction of a clinical epidemiologist. Information on disease burden and service readiness was collected from all facilities with regard to hypertension, DM, asthma, chronic obstructive pulmonary disease (COPD), heart failure, epilepsy and HIV infection. To assess individual health workers' levels of experience and service readiness for NCDs, we focused on hypertension and DM and compared this information with that on HIV infection. Three data collection tools were used:

- Service statistics on disease burden: Information was collected from facility records to extract the number of outpatients and the numbers of facility visits made by these patients specific to the selected CDs, as well as the number of all outpatient visits. These data were extracted for 3* *months from July to September 2012, that is during the dry season when access to health facilities was undisturbed by weather conditions.
- Service readiness: An adapted version of the WHO Service Availability and Readiness Assessment (SARA) questionnaire 15, 16, was used to collect data on facility characteristics, staff contingents, service availability, referral systems, drugs and equipment, laboratory services, reporting systems, monitoring and supervision. To obtain this information, we interviewed heads of facilities or their delegates, or heads of specific sections at larger facilities. Guidelines, reports, equipment, drugs and laboratory supplies were inspected.
- Health worker proficiency: A self‐completed questionnaire was administered anonymously to assess health workers' knowledge, experience, training, supervision, comfort level with case management, and perceived availability of drugs and equipment. Basic knowledge was assessed using case scenarios. The questionnaire was developed for this project, has been used in Tanzania and is available online 8.

All data collection tools were pre‐tested at two HCIIIs from Entebbe Municipality that did not take part in the main study.

### Analysis

We identified 10 study outcomes: one for current service provision, three for availability of guidelines and supplies, three for management and training systems and three for preparedness of human resources (Table 2). In addition, we assessed the number of staff at each facility in comparison with recommended staffing levels.

**Table 2:** Study outcome derivations

	Outcome	Form on which data collected	Derivation
Service provision			
1	Number and proportion of outpatient visits related to each chronic disease	Service statistics	Averaged across July–September 2012 and types of facilities
Availability of guidelines and supplies			
2	Availability of guidelines	SARA questionnaire	Guidelines observed in the outpatient clinic or respective specialist clinic for HIV, HTN or DM
3	Availability of basic diagnostic equipment	SARA questionnaire	HIV: Screening and confirmatory rapid tests available and not expired HTN: Digital blood pressure apparatus (or manual blood pressure apparatus AND stethoscope) observed and functioning in OPD or HTN clinic DM: Glucometer observed and functioning in OPD or DM clinic, or available and mostly/always functioning in laboratory (with test strips never or only occasionally out of stock)
4	Availability of first line drug therapy	SARA questionnaire	HIV: At least one‐first line regimen available and not expired (TDF + 3TC or TDF + FTC or AZT + 3TC or d4T + 3TC AND NVP or EFV) HTN: At least one thiazide diuretic (hydrochlorothiazide or bendrofluazide) available and not expired DM: Metformin available and not expired
Management and training systems			
5	Training	SARA questionnaire	Any outpatient staff member having received training in the diagnosis and management of HIV, HTN or DM within the last 2 years
6	Supervision	SARA questionnaire	Having received a monitoring or supervisory visit from a higher level of the health service within last 3 months for HIV, HTN or DM (asked of non‐communicable diseases combined and assumed to apply equally to HTN and DM)
7	Outreach	SARA questionnaire	Clinical outreach periodically carried out specifically to target HIV, HTN or DM
Preparedness of human resources			
8	At least fair knowledge	Self‐completed questionnaire	Assessed via case scenario questionnaires for HIV, HTN and DM, and defined as scoring at least 7/10
9	Experienced	Self‐completed questionnaire	Having seen ≥5 patients with each of HIV, HTN or DM in last 3 months
10	Comfortable	Self‐completed questionnaire	Reporting ‘very comfortable’ when asked whether feel comfortable with HIV, HTN or DM and know how to manage

HTN, hypertension; DM, diabetes mellitus; OPD, outpatient department; TDF, tenofovir; FTC, emtricitabine; 3TC, lamivudine; AZT, zidovudine; d4T, stavudine; NVP, nevirapine; EFV, efavirenz.

We summarised our findings as frequencies and percentages and compared outcomes by facility level or health worker cadre using Fisher's exact test for 2xc tables. When comparing health worker responses, we used the Pearson chi‐squared statistic with the second‐order correction of Rao and Scott to account for the clustering of health workers within facilities. To compare results related to hypertension and DM *vs*. HIV infection, we used sign tests to take account of matched responses within health facility or by health worker. All analyses were conducted using Stata version 12.

### Ethical approval

Approval was obtained from the ethics committees of the Uganda Virus Research Institute and the London School of Hygiene and Tropical Medicine, and from the Uganda National Council of Science and Technology. We also received clearance from the MOH and district health offices. These authorities contributed to the implementation of the study and the interpretation of the results. Information from individual health workers was collected anonymously after obtaining written informed consent.

## Results

### Health facility enrolment

Twenty‐eight facilities were selected and all agreed to participate. Of these, 86% (24/28) were public facilities, whilst four were PNFP faith‐based facilities. In these facilities, 222 (76%) of 292 eligible health workers were present and consented to be interviewed. The remainder were absent, and there were no refusals. The health workers who were interviewed consisted of 13 MDs (54% of all eligible), 29 non‐MD clinicians (93%), 121 nurses (82%) and 59 nurse assistants (66%). Two of the nurse assistants were laboratory assistants by training.

### Outpatient visits for chronic diseases and referral patterns

**Figure 1:**
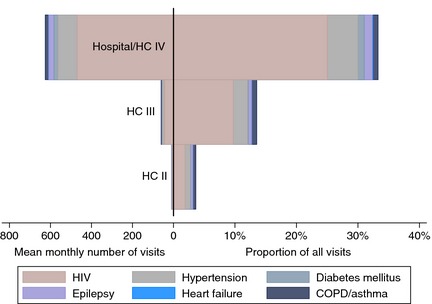
Burden of chronic diseases (CD) at 28 health facilities in Uganda: mean number of chronic disease visits per month per facility is displayed to the left of the midline and the proportion of all outpatient visits due to CD to the right. COPD, chronic obstructive pulmonary disease.

Hospitals and HCIVs recorded many more patients with CD than facilities at other levels (Figure 1 and Table 3). CDs contributed to a third of all outpatient visits at hospitals or HCIVs compared with 14% at HCIIIs and 4% at HCIIs. HIV was the most common CD in outpatient visits at all levels, accounting for 75% of CD visits at hospitals/HCIVs, 71% at HCIIIs and 56% at HCIIs. Hypertension and DM accounted for 15% and 3% of CD visits, respectively, at hospitals/HCIVs; 17% and 1% at HCIIIs, and 22% and <1% at HCIIs. Two percent of CD visits at hospital/HCIV level, 5% at HCIII and 11% at HCII were due to COPD or asthma.

**Table 3:** Burden of chronic diseases (CD) at 28 health facilities in Uganda: service statistics for July–September 2012, by health facility type

	Hospitals/Health centre IVs (*N* = 4)	Health centre IIIs (*N* = 12)	Health centre IIs (*N* = 12)
All visits/month/facility	1876	436	233
Hypertension			
Patients/month	89	9	2
Visits/month (% of all visits)	94 (5.0)	10 (2.3)	2 (0.9)
(% of CD visits)	(15.0)	(16.9)	(22.2)
Diabetes mellitus			
Patients/month	19	<1	<1
Visits/month (% of all visits)	19 (1.0)	<1 (0.1)	<1 (<0.1)
(% of CD visits)	(3.0)	(0.7)	(0.3)
Heart failure			
Patients/month	2	<1	0
Visits/month (% of all visits)	2 (0.1)	<1 (0.1)	0
(% of CD visits)	(0.3)	(0.4)	
COPD or asthma			
Patients/month	14	3	1
Visits/month (% of all visits)	15 (0.8)	3 (0.7)	1 (0.4)
(% of CD visits)	(2.4)	(5.1)	(11.1)
Epilepsy			
Patients/month	25	2	<1
Visits/month (% of all visits)	25 (1.3)	2 (0.5)	<1 (0.4)
(% of CD visits)	(4.0)	(3.4)	(9.6)
HIV infection			
Patients/month	386	39	5
Visits/month (% of all visits)	470 (25.1)	42 (9.6)	5 (2.1)
(% of CD visits)	(75.2)	(71.2)	(55.6)
Visits for CDs/month/facility	625	59	9
(% of all visits)	(33.3)	(13.5)	(3.9)

Results represent the mean number of patients and visits per month per facility, and proportion of visits as percentage of all visits made by patients to these facilities, and as a proportion of all visits made for CD.

COPD, chronic obstructive pulmonary disease.

All hospitals and HCIVs reported that they routinely managed patients with HIV, hypertension and DM. However, 50% (6/12) of HCIIIs routinely referred patients with HIV infection, 17% (2/12) referred cases with hypertension and 92% (11/12) referred those with DM. All HCIIs (12/12) reported that they routinely referred patients with these diseases. For all three diseases, the differences in referral routines between health facilities were highly significant (*P* < 0.001). Among the facilities routinely referring patients, very few reported receiving back‐referrals (3/18, 2/13 and 2/23 for HIV, hypertension and DM, respectively).

### Availability of guidelines and basic supplies

Guidelines for the management of HIV infection, hypertension and DM were seen in all hospitals/HCIVs. However, only 9/12 HCIIIs and none of the HCIIs had guidelines for HIV; and only 9/12 HCIIIs and 9/12 HCIIs had them for hypertension and DM (Table 4).

**Table 4:** Availability of guidelines and basic supplies and strength of management, training and reporting systems to ensure quality care for HIV, hypertension and diabetes mellitus at 28 health facilities in Uganda, by health facility level

Outcome	Disease	Hospitals and health centre IVs (*N* = 4) (%)	Health centre IIIs (*N* = 12) (%)	Health centre IIs (*N* = 12) (%)	*P*‐value (comparing levels of facilities)	Total (*N* = 28) (%)	*P*‐value (*vs*. HIV)^a^
Availability of guidelines and basic supplies							
Guidelines	HIV	4 (100)	9 (75)	0 (0)	<0.001	13 (42)	–
	HTN	4 (100)	9 (75)	9 (75)	0.69	22 (79)	0.01
	DM	4 (100)	9 (75)	9 (75)	0.69	22 (79)	0.01
Basic diagnostic equipment	HIV	4 (100)	11 (92)	4 (33)	0.007	19 (68)	–
	HTN	4 (100)	11 (92)	8 (67)	0.31	23 (82)	0.29
	DM	4 (100)	5 (42)	1 (8)	0.003	10 (36)	0.004
First‐line therapy	HIV	4 (100)	8 (67)	0 (–)	<0.001	12 (43)	–
	HTN	4 (100)	8 (67)	1 (8)	0.001	13 (46)	>0.99
	DM	3 (75)	2 (17)	0 (–)	0.006	5 (18)	0.07
Strength of management, training and reporting systems							
Training of any HW past 2 years	HIV	4 (100)	8 (67)	8 (67)	0.63	20 (71)	–
	HTN	2 (50)	0 (0)	0 (0)	0.02	2 (7)	<0.001
	DM	2 (50)	0 (0)	0 (0)	0.02	2 (7)	<0.001
Supervision received last month	HIV	4 (100)	10 (83)	4 (33)	0.02	18 (64)	–
	HTN	1 (25)	1 (8)	2 (17)	0.79	4 (14)	<0.001
	DM	1 (25)	1 (8)	2 (17)	0.79	4 (14)	<0.001
Outreach	HIV	4 (100)	9 (75)	9 (75)	0.69	22 (79)	
	HTN	2 (50)	3 (25)	3 (25)	0.72	8 (29)	<0.001
	DM	2 (50)	1 (8)	1 (8)	0.13	4 (14)	<0.001

See Table 2 for outcome definitions. HTN, hypertension; DM, diabetes mellitus.

a From sign tests to take account of matched responses within health facility.

All four hospitals/HCIVs had basic diagnostic tools (Table 4) for hypertension, DM and HIV infection. Only 5/12 HCIIIs and 1/12 HCII had these for DM (*P* = 0.003). Rapid HIV test kits were available at 11/12 HCIIIs but only at 4/12 HCIIs (*P* = 0.007). There was no evidence of a significant difference across facility levels in the availability of basic equipment for the diagnosis of hypertension (*P* = 0.31). However, one HCIII and 4/12 HCIIs had no functioning blood pressure machine. Overall, 12/24 and 19/24 HCIIIs and HCIIs reported that they lacked functioning weighing scales and height measurement devices, respectively. 11/23 HCIIIs and HCIIs reported using urine dipsticks for glucose screening but only 5/23 reported a consistent supply of these dipsticks without stock‐outs.

All hospitals/HCIVs had first*‐*line drugs (Table 4) for HIV infection and hypertension available; however, one did not have metformin, the first*‐*line drug for treatment of DM. Only 8/12 HCIIIs had first*‐*line antiretroviral drugs (ARVs) for HIV infection, whereas two of those four HCIIIs without first*‐*line ARVs were ARV‐accredited at the time. None of the HCIIs had ARVs, in line with current MOH 2012 guidelines for this level. Cotrimoxazole, for prophylaxis of infections in HIV*‐*infected patients, was available at all four hospital/HCIVs, 8/12 HCIIIs and 6/12 HCIIs. First‐line drugs for treatment of hypertension were available at only 8/12 HCIIIs and one HCII (*P* = 0.001), although these facilities should stock these drugs according to current MOH 2012 guidelines. First*‐*line therapy for DM was available at 2/12 HCIIIs and 0/12 HCIIs (*P* = 0.006).

Drug stock‐outs were commonly reported by all health facilities. In total, 22/28 of facilities reported experiencing stock‐outs at least occasionally; 3/12 HCIIIs and 4/12 HCIIs experienced this often or always.

### Management and training

Overall training, supervision and community outreach were weaker for hypertension and DM than for HIV (Table 4). Training of at least some staff on HIV case management within the last 2 years was reported by all hospitals/HCIVs, 8/12 HCIIIs and 8/12 HCIIs (*P* = 0.63, comparing facility levels), while training for either hypertension or DM was reported by 2/4 hospitals/HCIVs and none of the HCIIIs or HCIIs (*P* = 0.02).

Having received support supervision visits within the last month for HIV case management was reported by 18/28 facilities compared with 4/28 for either hypertension or DM. All four hospitals/HCIVs reported supervision visits for HIV case management compared with 10/12 HCIIIs and 4/12 HCIIs (*P* = 0.02). Only one hospital/HCIV, one HCIII and two HCIIs reported having had support supervision for either hypertension or DM (*P* = 0.79).

Regular outreach activities were reported by all facilities except one (data not shown). These 27 had periodically conducted outreach related to health promotion, and 20 reported outreach to conduct case finding for specific diseases. HIV infection was the most frequently targeted CD by outreach services at all levels (79% of all health facilities, Table 4). Hypertension was the second most frequently targeted CD, but outreach occurred significantly less frequently than for HIV infection, at 29% of all health facilities (*P* < 0.001). DM was targeted by only 14% of all health facilities with only 1/12 HCIIIs and 1/12 HCIIs reporting such services (*P* < 0.001 compared with HIV).

Among the 27 health facilities conducting outreach, various obstacles were commonly reported: insufficient staff/time (26/27), insufficient funds (25/27), lack of transport (22/27), staff insufficiently trained (21/27), lack of drugs (19/27) and lack of equipment (16/27).

### Preparedness of human resources

Overall, 184 (83%) of the 222 health workers who completed the individual questionnaire demonstrated fair knowledge of HIV case management, compared with 72% for hypertension (*P* < 0.001) and 52% for DM (*P* < 0.001) (Table 5). There was no evidence of a significant difference between facility levels in health worker knowledge of HIV (*P* = 0.28); however, there was some evidence that knowledge of hypertension (*P* = 0.06) and DM (*P* = 0.02) varied by level of facility, with knowledge being lowest at the lowest facility level. For example, only 31% of HCII staff had fair knowledge of DM, compared with 51% at HCIIIs and 60% at hospital/HCIV. Not surprisingly, MDs were generally more knowledgeable on all three CDs than non‐MD clinicians, nurses and nurse assistants (*P* = 0.02 for knowledge of HIV, *P* < 0.001 for hypertension or DM).

**Table 5:** Current level of preparedness of human resources to ensure quality care for HIV, hypertension and diabetes mellitus at 28 health facilities in Uganda, among 222 healthcare workers by: (A) health facility level and (B) healthcare worker cadre

Outcome	Disease	Hospitals/health centre IVs (*N* = 103) (%)	Health centre IIIs (*N* = 83) (%)	Health centre IIs (*N* = 36) (%)	*P*‐value (comparing levels of facilities)*	Total (*N* = 222) (%)	*P*‐value (*vs*. HIV)†
At least fair knowledge	HIV	81 (79)	73 (88)	30 (83)	0.28	184 (83)	–
	HTN	75 (73)	64 (77)	20 (56)	0.06	159 (72)	<0.001
	DM	62 (60)	42 (51)	11 (31)	0.02	115 (52)	<0.001
Experienced	HIV	89 (86)	72 (87)	16 (44)	<0.001	177 (80)	–
	HTN	49 (48)	23 (28)	5 (14)	0.003	77 (35)	<0.001
	DM	30 (29)	7 (8)	0 (0)	0.02	37 (17)	<0.001
Comfortable	HIV	28 (27)	15 (18)	4 (11)	0.16	47 (21)	–
	HTN	21 (20)	5 (6)	0 (0)	0.02	26 (12)	0.002
	DM	13 (13)	2 (2)	1 (3)	0.02	16 (7)	<0.001

Outcome	Disease	MDs (*N* = 13) (%)	Non‐MD clinicians (*N* = 29) (%)	Midwives (*N* = 34) (%)	Nurses (*N* = 87) (%)	Assistants (*N* = 59) (%)	*P*‐value (comparing health worker cadres)*
At least fair knowledge	HIV	13 (100)	28 (97)	31 (91)	74 (85)	38 (64)	0.02
	HTN	13 (100)	29 (100)	21 (62)	70 (80)	26 (44)	<0.001
	DM	13 (100)	24 (83)	10 (29)	52 (60)	16 (27)	<0.001
Experienced	HIV	12 (92)	28 (97)	27 (79)	70 (80)	40 (68)	0.009
	HTN	10 (77)	18 (62)	6 (18)	28 (32)	15 (25)	0.001
	DM	6 (46)	10 (34)	0 (0)	14 (16)	7 (12)	<0.001
Comfortable	HIV	6 (46)	9 (31)	3 (9)	22 (25)	7 (12)	0.008
	HTN	11 (85)	5 (17)	0 (0)	7 (8)	3 (5)	<0.001
	DM	8 (62)	3 (10)	0 (0)	4 (5)	1 (2)	<0.001

See Table 1 for outcome definitions. HTN, hypertension; DM, diabetes mellitus; MD, medical doctor.

Definition of ‘fair knowledge’: health worker scored at least 7/10.

Definition of ‘Experienced’: managed at least five cases over last 3 months.

*Rao‐Scott F (second‐order correction to the Pearson *χ* ^2^ statistic to account for clustering of health workers within facilities); †From sign tests to take account of matched responses within health worker.

Experience of health workers in having managed at least five cases of a specific CD during the last 3 months was much lower for hypertension (35%) and DM (17%) than for HIV infection (80%; *P* < 0.001). Experience of CD management was significantly lower at lower‐level health facilities (*P* < 0.001, 0.003 and 0.02 for HIV, hypertension and DM, respectively). For example, 48% of healthcare workers at hospitals reported having managed five patients or more with hypertension in the last 3 months compared with only 28% and 14% at HCIIIs and HCIIs, respectively.

**Figure 2:**
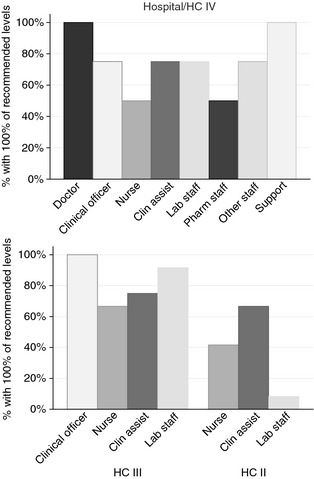
Staffing levels at 28 health facilities in Uganda: proportion of facilities having 100% of the recommended staffing levels for each cadre of healthcare worker, by health facility level.

Assessment of staffing levels showed that none of health facilities had all the staff recommended according to MOH guidelines (Figure 2) 11. HCIIs were particularly affected by staff shortages. The greatest shortage was observed with respect to nurses: only 2/4 hospitals and HCIVs 8/12 (65%) HCIIIs and 5/12 (41%) HCIIs had all nursing staff positions filled. Among those facilities that had a shortage of nurses, nursing staff reached 54%, 67% and 36% of recommended levels in hospitals/HCIVs, HCIIIs and HCIIs, respectively. The situation was better with respect to staffing levels of doctors at hospitals and HCIVs and of clinical officers at HC IIIs, where all facilities surveyed had the recommended level of staff.

## Discussion

In this representative sample of health facilities from two districts of central Uganda, most patients with CDs were seen at hospitals and HCIVs. Considerably fewer patients with CD were recorded at HCIIIs and very few at HCIIs, although these facilities are expected to diagnose and manage uncomplicated CD cases. This was also the case in rural areas for which HCIIIs and HCIIs are the backbone of health care in Uganda. The population burden of NCDs in rural Uganda is high 17, 18, 19. In our own survey of a representative sample of 889 adults living in urban and rural areas surrounding the health facilities described in this study, the prevalence of hypertension was 25% and of DM was 3%, with similar prevalence in urban and rural areas 14. Half of those with hypertension had not had their blood pressure measured in the past 5* *years, and 42% had never had their blood pressure measured (unpublished data Kavishe B, Biraro S, Baisley K, Vanobberghen F, Kapiga S, Munderi P, Smeeth L, Peck R, Mghamba J, Mutungi G, Ikoona E, Levin J, Plus AB, Katende D, Kisanga E, Hayes R, Grosskurth H.) One third of those with diabetes had not had their glucose levels measured in the past 12* *months. This means that currently many patients with these conditions are not seeking treatment. Our survey of health facilities suggests that those who do seek treatment at lower*‐*level facilities are referred to higher levels without attempting to diagnose or treat. In consequence, hospital OPDs may be overburdened with uncomplicated CD cases, whilst lower‐level health facilities are underutilised.

The underutilisation of lower‐level health facilities for CD care is likely due to a variety of problems, including a lack of guidelines, diagnostic tools and first*‐*line drugs. This was compounded by a lack of training and support supervision at these facilities, and a lack of experience in managing CDs, in particular NCDs. Researchers in other parts of Africa have made similar observations, indicating that Uganda is not unique in this respect 6, 8. This suggests a general discrepancy between the large and increasing burden of CDs, particularly NCDs, and the ability of health systems in the region to cope with it. Interventions to overcome these shortcomings through training, improved logistics and better health system management are likely to also improve service uptake and lead to a reduction in the burden of routine cases at hospitals 19, 20.

To achieve these objectives, much can be learned from the ongoing HIV control activities in Uganda. Over the last two decades, Uganda successfully expanded HIV services to lower levels of health care 21, 22, as also reflected in our study in which we saw a much higher level of service readiness for HIV infection than for other CDs. These findings imply that NCD services, like HIV services, could also be provided effectively at lower levels of health care. Encouragingly, health workers of all cadres and from all types of facilities expressed interest during interviews in learning more about NCD case management.

We identified other strengths in the Ugandan health system that could be used to improve CD services at smaller facilities. First, the many highly experienced hospital‐based health workers could play a stronger role in providing training and supervision for health workers at smaller facilities. Second, because most facilities regularly conduct some kind of outreach services, activities for health promotion and NCD prevention could be provided to communities as part of these existing outreach services. The effectiveness of such programmes has been shown elsewhere in Uganda 23 and is in line with the WHO action plan on NCDs 24.

None of the observed levels of health care had the recommended norms (100%) for nursing staff and in particular many health facilities at lower levels of health care. The availability of sufficient human resources is important for the management of CDs. However, for HCIIs, the availability of nurses is critical as these facilities are usually run by a nurse. The growing burden of NCDs will require similar efforts towards task shifting as have been made for HIV control in Uganda, but this will only be effective if the required human resources are available and sufficiently supported 21.

The necessary efforts to strengthen lower‐level health facilities will cost some money, for example small*‐*scale investments in basic equipment such as blood pressure machines and brief refresher trainings of existing staff. Recurrent costs would also have to increase, for example, by bringing staff contingents in health facilities to the officially recommended levels, although this should be largely covered through current budgets; and importantly by increasing the intensity of supportive supervision, at least for some time. Additional expenditures to meet the increasing demand for essential NCD drugs will be inevitable.

Our study has a number of strengths. First, we looked at different aspects of health service readiness using several complementary methods which allowed us to generate a comprehensive and robust assessment. Second, our data were collected using standardised questionnaires which had been pilot‐tested and used elsewhere. To assess service readiness, we applied an adapted version of the WHO SARA questionnaire. This tool is increasingly being employed in other resource limited countries, making our data comparable with other studies 16. Finally, the study was embedded in a wider research programme to investigate the burden and establish interventions for CDs in Uganda and Tanzania, allowing us to triangulate data on the burden of CDs observed at health facilities with information from a population‐based survey 14. Our population survey indicated that the burden of NCDs is higher than that seen in the health facilities and that many people with NCDs are not seeking care for their conditions 14. Therefore, the demand for NCD care at health facilities is likely to rise, making further improvements of health services even more urgent.

Our study has some limitations. Although we included a representative sample of health facilities, our data refer to two districts of Uganda only; therefore, it is not clear how generalisable our results may be to other parts of the country. However, a co‐investigator of this study (GM), from the NCD department at the MOH, confirmed that our findings are consistent with the situation of the health services elsewhere in Uganda. Secondly, although we aimed to interview all health workers who were involved routinely in CD management, some staff were absent on the days that we conducted the interviews so could not be reached. Overall, we were able to interview 76% of eligible staff, with no refusals. However, the coverage varied by health worker cadre: we interviewed 82% of nurses and 93% of non‐MD clinicians, but only 54% of MDs. It is possible that the MDs that we interviewed were not representative of all MDs, and so some selection bias may have occurred. Lastly, as this was a descriptive study, all *P*‐values reported were intended to be exploratory rather than to assess hypotheses. No adjustments were made for multiplicity of testing. Moderately significant *P*‐values should be interpreted with caution.

## Conclusion

In conclusion, the healthcare system of Uganda, as that of other countries from SSA, is currently not coping with the increasing burden of CDs and in particular NCDs. Major improvements are needed, particularly at lower‐level health facilities (HCIIIs and HCIIs) that are situated in areas where many patients with NCDs live. This is urgent because higher levels of health care are overburdened with the management of routine cases. Improvements should be geared towards three main areas: (i) provision of standard guidelines, essential diagnostic equipment and drugs; (ii) training of healthcare workers, follow‐on supervision and adapted referral procedures; and (iii) ensuring that a critical minimum number of staff is in place at peripheral health facilities. Service readiness for basic HIV care was substantially better at all levels, demonstrating that improvements in NCD care are feasible. The achievements of the Uganda health system in HIV care could serve as a model for these efforts. The strengths of the system, for example highly experienced healthcare workers from hospitals, existing outreach activities and the desire of health workers to expand services, provide an opportunity that can be built upon.
